# An Effective Protocol for Management of International Arrivals at Risk in COVID-19 Pandemic: Experience From the Pre-Hospital Covid-19 Response Teams at Xi'an, China

**DOI:** 10.3389/fpubh.2022.753640

**Published:** 2022-03-07

**Authors:** Yifan Zou, Yuliang Zou, Anthony M. Dart, Yuping Zhang, Yousen Wang, Fenling Fan

**Affiliations:** ^1^School of Economics and Finance, Xi'an Jiaotong University, Xi'an, China; ^2^Department of Gynecology and Obstetrics, The First Hospital of Xi'an Jiaotong University, Xi'an, China; ^3^Office of Medical Administration, The First Hospital of Xi'an Jiaotong University, Xi'an, China; ^4^Baker Institute & Cardiovascular Medicine, The Alfred, Melbourne, VIC, Australia; ^5^Department of Cardiovascular Medicine, The First Hospital of Xi'an Jiaotong University, Xi'an, China

**Keywords:** COVID-19, travel, China, transmission, control

## Abstract

**Background:**

The coronavirus disease 2019 (COVID-19) outbreak within China has been well controlled and stabilized since early April 2020. Therefore, the current major focus in China is to prevent the introduction of COVID into China from international arrivals. To achieve this, pre-Hospital COVID-19 Response Teams (pHCRTs) have been established.

**Context:**

The pHRCTs were established in Xi'an, China in early 2020. During the 12 months covered in this report, there were 356 international flight arrivals with over 5,000 COVID-19 Nucleic Acid Test (NAT) positive people, 500 of them with symptomatic COVID-19 and requiring admission to special hospitals. All other arrivals were managed in dedicated facilities by pHRCTs. The outcome measure of this report was the number of positive cases among the pHRCT members.

**Details:**

Four hundred forty-two staff worked in the pHCRTs during the reporting period. Despite multiple throat swab PCR tests during their pHRCTs tour of duty, and the subsequent mandatory 14-day quarantine required before return to the general community, no staff became NAT positive.

**Conclusion:**

The prevention of community transmission from imported cases is a vital part of the strategy to maintain the low numbers of cases in countries which have achieved control, or suppression of local internal cases. The program of pHCRTs described in this article gives successful protocols for transportation of patients who are infectious based on the minimal transmission of virus and staff safety. The strategies employed may prove useful in future pandemics.

## Introduction

The coronavirus disease 219 (COVID-19) outbreak within China has been well controlled and stabilized since early April 2020, with less than 10 locally occurring cases reported daily since mid-March (1). This success in controlling the local transmission has allowed the restoration of social and commercial life almost to a pre-COVID-19 state. However, the risk of imported cases, with a consequent resurgence in domestic cases persist (2). Thus, effective management of international arrivals is of paramount importance. Most of such arrivals in China come by air. While China has many airports capable of receiving international flights, 12 of them were designated as transfer cities for airlines to Beijing to facilitate an effective strategy to prevent introduction of the virus into Beijing. A further strategy was to limit or postpone flights from countries when previous flights presented a particularly high positive COVID-19 caseload.

Xi'an City, with a population of 10 million (3), is the biggest City in North-west China and, pre-COVID-19, annually received approximately 4.7 million international arrivals (4). To deal with anticipated problems in controlling the risk from infected arrivals, pre-Hospital COVID-19 Response Teams (pHCRTs) were built up from January 2020. This has allowed all hospitals to continue with their normal workload while, at the same time, providing a suitably qualified. To date, no member of the pHCRTs have become infected with COVID-19 despite the arrival of over 5,000 COVID-19 positive international arrivals during this period. In view of this success, we believe this experience will be of value to other countries in dealing with this and with future pandemics, which are generally accepted as likely to occur in the future.

## Context

### Organization

**Figure 1:**
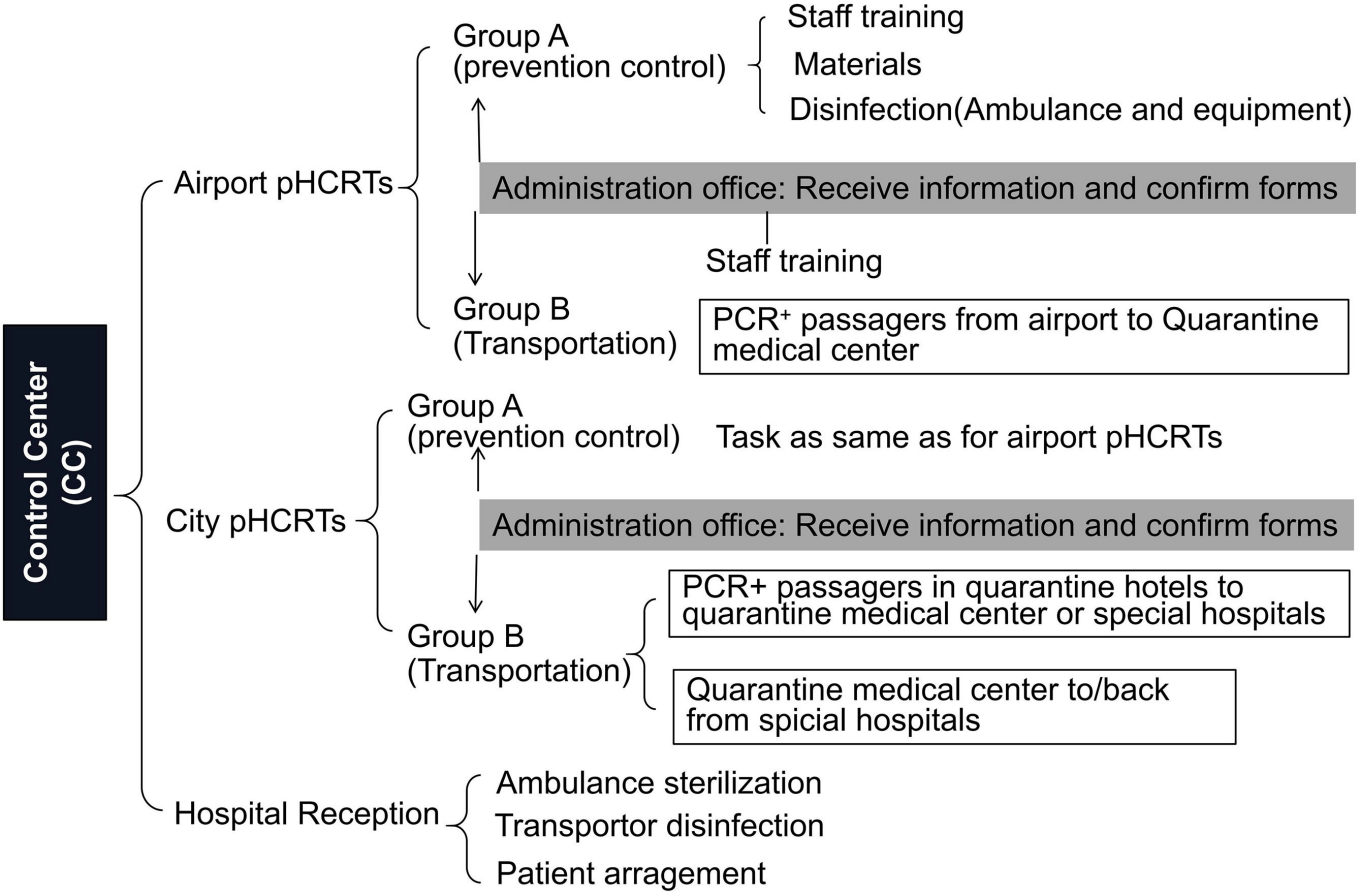
Organization and tasks of pre-hospital COVID-19 response teams (pHCRT) groups. Task for group A in open writing; task for group B in writing with square; task for administrators in writing with shadow.

**Figure 2:**
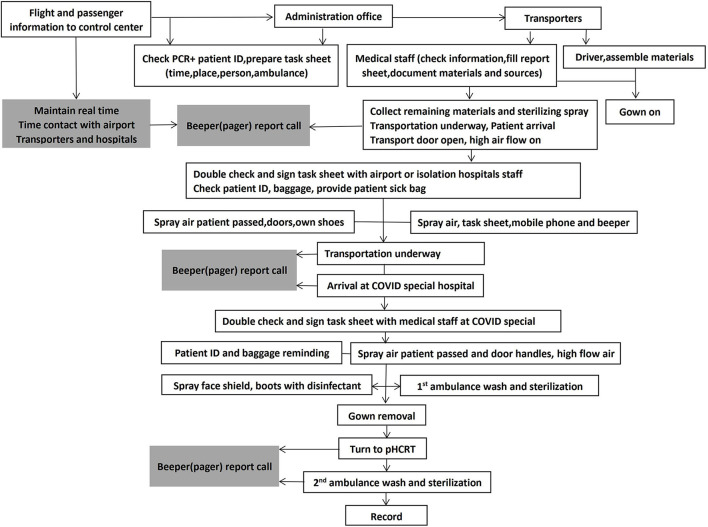
Flowchart of transportation work of Group B. Task for Group B in writing with square; task for administrators in writing with shadow.

Pre-Hospital COVID-19 Response Teams (pHCRTs) were organized by Xi'an City medical bureau based on the Pre-hospital Emergency Control Center initially established in 1952 and, by 2012, comprising a network of 37 emergency substations and 50 rural sites. The pHCRTs comprise 24–36 dedicated ambulances. Each ambulance is staffed with either a nurse or a doctor together with one driver from 27–31 medical institutes or hospitals. An administrative office communicates between pHCRTs, Control Center, staff training, and quality control (Figures 1, 2). Three pHCRTs of 8–12 ambulances were assembled. Two pHCRTs were based in the Northern part of the city, close to both entrance of freeways to airports, and COVID quarantine centers in addition to the COVID special hospitals or units in general hospitals. The remaining pHCRT is based at the airport. Each pHCRT is divided into 2 groups. Group A is in charge of materials, disinfection, and staff training, while group B (transporters) is assigned to transport suspected or diagnosed patients (Figure 1). The process is coordinated from a Control Center in continual real-time communication with the site administration offices and medical transporters of the three pHRCTs (Figure 2).

### Equipment and Process

Dedicated ambulances are used to transport COVID PCR positive passengers between the airport and quarantine hotel to the medical quarantine center and between the quarantine center and hospitals for those who require specialized treatment. The driver and nurse or doctor sit in the compartment of the driver. Patients travel in the rear section which is equipped with suction, stretcher, oxygen, call button, and first aid equipment. In addition, the rear compartment has a powered high-flow air throughput.

Each vehicle has a bag with 11 items (single-use boots, gloves, Caps, Gowns, N95 masks) prepared by group A (prevention control) and used throughout the whole transport process by Group B. In addition, there are sterilizers for hands, mobile phones, pagers, seats, and task sheets of transporters when these have been exposed to the risk of possible contamination. Medical staff always have a sterilizing spray to sterilize all parts of the vehicle when patients enter or exit the vehicle. Staff shoes are sprayed prior to re-entry to the vehicle. The drivers remain seated throughout the process but help spray the front compartment, medical staff hands, and items used during patient transport, including task sheet, mobile, beeper, and holder of spray.

Two aspects receive particular attention during staff training and process implementation by the transport staff in group B. The first is disinfection and sterilization of vehicles and equipment. The second is putting on and removing the protective clothing (gowns, gloves, face shields, etc.). The first includes spraying air around patients during their entry to and exit from ambulances and transport vehicles. Air is sprayed with liquid “84 sterilization liquid” (mainly sodium hypochlorite 500 mg/L, made in China). Full disinfection and sterilization of ambulances are undertaken 3 times each day, either with 84 sterilization liquid or with 7.5% H_2_O_2_ liquid in CLEANCube containers with spray arms (10 ml/m^3^). Disinfection of transporter staff equipment, including face shield, gown, boots, and shoe soles, was undertaken by spraying 500 mg/L 84 sterilization liquid from a car wash. A mandatory 7-step hand wash procedure was utilized and required at each step of gown and boot removal.

### Details

Since the start of the program, there were 438 staff, aged from 21–68 years old (mean 35), including 110 doctors, 142 nurses, 179 drivers, 6 administrators, and 1 cleaner. All were volunteers and required a negative COVID PCR test and an assessment of their previous 14-day travel history prior to being accepted into the program. The mean duration of pHCRTs work for each of these groups was 62 days, 68 days, 96 days, and 334 days.

During this period, staff remained isolated from the general community. Staff members submitted a daily health questionnaire, had their temperature monitored at every building and vehicle entry, and had a COVID-19 PCR test every 7 days. Driving records were submitted after each journey, detailing the vehicle passengers and staff. At the end of the secondment period, each staff member undergoes a 2-week quarantine before returning to his or her base institute and the general community. In addition, they require 3 negative COVID-19 PCR tests, conducted every 1st day of a continuous 3-week period.

According to the data from the administration office, there were 356 international flights during the reporting period (until the end of 2020) (5). All arrivals required either a throat swab or a blood test. Patients from high-risk countries required both. From these flights, there were over 5,000 COVID-19 Nucleic Acid Test (NAT) positive people. In total 500 of these 5,000 had symptomatic COVID requiring admission to specialized hospitals. All other arrivals were managed in dedicated facilities by pHCRTs. No staff of pHCRTs has become NAT positive as evidenced from the multiple negative throat swab PCR conducted during their period of secondment and subsequent 2 weeks of self-quarantine.

## Discussion

Coronavirus disease 2019 (COVID-19), a respiratory disease caused by the SARS-CoV-2 virus, was declared a pandemic by the WHO on March 11, 2020. The rapid spread of the disease has taken the scientific and medical community by surprise and has presented unique challenges to International public health due to its relative ease of transmission (now increased by the development of new strains, such as Delta and Omicron) and significant rates of morbidity and mortality. Both pre-symptomatic and asymptomatic transmissions have been described and are likely to play important roles in the dynamics of the pandemic (6). In addition, the ubiquity of international travel, particularly over long distances by air, has greatly facilitated this spread. Even as early May 2020, more than 5 million people had been infected with the virus, causing more than 330,000 deaths in over 210 countries (7). Furthermore, there are recurrent waves of infection (8). Recent data indicates approximately 270 million cases and 5 million deaths worldwide by December 12, 2021 (9). Unfortunately, while supportive measures and treatment have been reduced in hospital mortality, there is a lack of specific drug treatment and limited vaccine availability. Therefore, a range of non-pharmacological interventions has been put into place by governments to contain and mitigate the spread of COVID-19. Despite the increasing availability of effective vaccines, principally, in developed countries, such measures will continue to play a critical role in containing the SARS-CoV-2 pandemic for a significant period of time into the future. Travel-related control measures range from the screening of travelers entering or leaving a country to the complete closure of national borders. Starting from February 2020, many countries and regions in the world have implemented some type of travel-related control measures. It is crucial to understand the effectiveness of these measures, including the preventive strategies for people who are physically close to infectious or diagnosed patients, such as medical staff or public service staff. It is possible that an undetected infection transmission among staff may have resulted in small-scale infection clusters, (10, 11) even when implementing the preventive standard recommended by the WHO guidelines (12) or used in the initial outbreak in Wuhan (13). The principal finding from our experience is that it is possible to manage international arrivals by air without any introduction of COVID-19 into the local community. Thus, despite over 5,000 arrivals positive for COVID and a rigorous testing regimen, we have not found a single case of COVID among the staff managing airport arrivals. This success has resulted from meticulous attention to a detailed management plan, and from the basic premise that effective quarantine and testing of the international arrivals, protection, and quarantine of front-line staff is of crucial importance to prevent exposure of the general community.

Following the initial outbreak in Wuhan, China has achieved effective suppression of locally acquired infection, such that future threats arise primarily from importation through international arrivals. The overwhelming majority of international arrivals are *via* international air travel. Hence, custom authorities introduced NAT for all overseas passengers entering China, combined with quarantine (14, 15). Travelers are subject to a 14-day mandatory quarantine in the first entry point city. At least 4–5 NATs are compulsory for all passengers (arrival at the airport, after 24 h at hotels or medical centers, 1 week, and 2 weeks after arrival) until they complete a fortnight quarantine period and test negative for the virus. The work of transportation of NAT positive cases, from the airport or between special hotels or medical centers to COVID hospitals or comprehensive hospitals with a COVID ward, has also been done only by the special pHCRTs. While Xi'an's pHCRTs are among the earliest and largest in China, they have successfully prevented any transmission to the front-line staff. This approach, together with early recognition and information sharing, will help prevent a similar situation from arising in a future pandemic.

Are there alternative or additional procedures? Complete cessation of international travel is not a realistic option. A more realistic option, particularly in concert with the measures described here, would be to require proof of COVID-19 status shortly before travel. Such measures are already in use in some jurisdictions. In view of the current, albeit limited, availability of an effective vaccination program, a requirement for proof of vaccination and evidence of no active infection may be realistic. However, even such a strategy may not be acceptable in view of the likely discrimination against less developed countries with limited access to vaccines and testing. In addition, some people will be unable to be vaccinated because of other health issues, so that effective procedures for the safe arrival of incoming international passengers will be required for the foreseeable future. In summary, we believe the processes we have described offer important lessons for the management of international arrivals not only under present circumstances, but also in any future pandemic.

Despite the increasing levels of immunization, principally in well-developed countries, the need to successfully prevent or limit the international spread of the virus remains a major strategy in allowing the world to begin to return to pre-COVID-19 conditions. The program of pHCRTs described in this article provides successful protocols for transportation of infectious patients with minimal onward transmission and maintained staff safety.

## Data Availability Statement

The original contributions presented in the study are included in the article/supplementary material, further inquiries can be directed to the corresponding author/s.

## Ethics Statement

Ethical review and approval was not required for the study on human participants in accordance with the local legislation and institutional requirements. Written informed consent for participation was not required for this study in accordance with the national legislation and the institutional requirements.

## Author Contributions

All authors listed have made a substantial, direct, and intellectual contribution to the work and approved it for publication.

## Funding

This study was supported by the Clinical Research Award of the First Affiliated Hospital of Xi'an Jiaotong University, China (No. XJTU1AF-CRF-2019-010) and Fundamental Project Plan in Shannxi Province, China (No. 2020JM-364).

## Conflict of Interest

The authors declare that the research was conducted in the absence of any commercial or financial relationships that could be construed as a potential conflict of interest.

## Publisher's Note

All claims expressed in this article are solely those of the authors and do not necessarily represent those of their affiliated organizations, or those of the publisher, the editors and the reviewers. Any product that may be evaluated in this article, or claim that may be made by its manufacturer, is not guaranteed or endorsed by the publisher.

## References

[1] Liu K Zhang W Yang Y Zhang J Li Y Chen Y. Respiratory rehabilitation in elderly patients with COVID-19: A randomized controlled study. Complement Ther Clin Pract. (2020) 39:101166. 10.1016/j.ctcp.2020.101166 32379637 PMC7118596

[2] Fang LQ Sun Y Zhao GP Liu LJ Jiang ZJ Fan ZW. Travel-related infections in mainland China, 2014-16: an active surveillance study. Lancet Public Health. (2018) 3:e385–94. 10.1016/S2468-2667(18)30127-0 30033200 PMC7164813

[3] Song YZ Yang HL Peng JH Song YR Sun Q Li Y. Estimating PM2.5 concentrations in Xi'an city using a generalized additive model with multi-source monitoring data. PLoS ONE. (2015) 10:e0142149. 10.1371/journal.pone.0142149 26540446 PMC4634950

[4] China Travel ccaoneline cn. etal. Xi'an Xianyang Airport: passenger throughput reaches 40 millions in (2017). Available online at: https://www.chinatravel.com/china-flights/xian-xianyang-airport.

[5] Xi'an international airport Baidu Pengbai Media. Passage throughput in Xi'an Xianyang International Airport (2020). Available online at: http://www.xxia.com/index.jsp. https://baijiahao.baidu.com/s?id=1679583134206770896&wfr=spider&for=pc. https://m.thepaper.cn/newsDetail_forward_11472550?ivk_sa=1024320u

[6] Furukawa NW Brooks JT Sobel J. Evidence supporting transmission of severe acute respiratory syndrome coronavirus2 while presymptomatic or asymptomatic. Emerg Infect Dis. (2020) 26:e201595. 10.3201/eid2607.201595 32364890 PMC7323549

[7] Ortiz-Prado E Simbaña-Rivera K Gómez- Barreno L Rubio-Neira M Guaman LP Kyriakidis NC. Clinical, molecular, and epidemiological characterization of the SARS-CoV-2 virus and the Coronavirus Disease 2019 (COVID-19), a comprehensive literature review. Diagn Microbiol Infect Dis. (2020) 98:115094. 10.1016/j.diagmicrobio.2020.115094 32623267 PMC7260568

[8] Strzelecki A. The second worldwide wave of interest in coronavirus since the COVID-19 outbreaks in South Korea, Italy and Iran: A Google Trends study. Brain Behav Immun. (2020) 88:950–1. 10.1016/j.bbi.2020.04.042 32311493 PMC7165085

[9] John E. COVID-19 cases and deaths among hardest hit countries worldwide as of Dec. 12, 2021. Health, Pharma & Medtech. State of Health. Available online at: https://www.statista.com/statistics/1105264/coronavirus-covid-19-cases-most-affected-countries-worldwide/. (accessed Dec 8, 2021)

[10] Ogawa F Kato H Sakai K Nakamura K Ogawa M Uchiyama M. Environmental maintenance with effective and useful zoning to protect patients and medical staff from COVID-19 infection. Acute Medicine & Surgery. (2020) 7:e536. 10.1002/ams2.536 32685174 PMC7300484

[11] Hibino M Iwabuchi S Munakata H. SARS-CoV-2 IgG seroprevalence among medical staff in a general hospital that treated patients with COVID-19 in Japan: retrospective evaluation of nosocomial infection control. J Hosp Infect. (2021) 107:103–4. 10.1016/j.jhin.2020.10.001 33039454 PMC7544697

[12] World Health Organization. Rational use of personal protective equipment for coronavirus disease (COVID-19) and considerations during severe shortages: interim guidance. Geneva: WHO, (2020). Available online at: https://www.who.int/publications/i/item/rationaluse-of-personal-protective-equipment-for-coronavirus-disease-(covid-19)-and-considerations-during-severe-shortages

[13] Liu M Cheng SZ Xu KW Yang Y Zhu QT Zhang H. Use of personal protective equipment against coronavirus disease 2019 by healthcare professionals in Wuhan, China: cross sectional study. BMJ. (2020) 369:m2195. 10.1136/bmj.m2195 32522737 PMC7284314

[14] Li XY Min Liu M Zhou R Zhang Y Wu CH Xu L. Centralized medical quarantine for imported COVID-19 in Shanghai, China. J. Travel Med. (2020) 27:taaa109. 10.1093/jtm/taaa109 32634217 PMC7529111

[15] Yan YC Chen H Chen LQ Cheng B Diao P Dong LY. Consensus of Chinese experts on protection of skin and mucous membrane barrier for health-care workers fighting against coronavirus disease 2019. Dermatol Ther. (2020) 33:e13310. 10.1111/dth.13310 32170800 PMC7228211

